# Cross-Frequency Coupling and Intelligent Neuromodulation

**DOI:** 10.34133/cbsystems.0034

**Published:** 2023-05-31

**Authors:** Chien-Hung Yeh, Chuting Zhang, Wenbin Shi, Men-Tzung Lo, Gerd Tinkhauser, Ashwini Oswal

**Affiliations:** ^1^ School of Information and Electronics, Beijing Institute of Technology, Beijing, China.; ^2^ Department of Biomedical Sciences and Engineering, National Central University, Taoyuan, Taiwan.; ^3^ Department of Neurology, Bern University Hospital and University of Bern, Bern, Switzerland.; ^4^ MRC Brain Network Dynamics Unit, University of Oxford, Oxford, UK.

## Abstract

Cross-frequency coupling (CFC) reflects (nonlinear) interactions between signals of different frequencies. Evidence from both patient and healthy participant studies suggests that CFC plays an essential role in neuronal computation, interregional interaction, and disease pathophysiology. The present review discusses methodological advances and challenges in the computation of CFC with particular emphasis on potential solutions to spurious coupling, inferring intrinsic rhythms in a targeted frequency band, and causal interferences. We specifically focus on the literature exploring CFC in the context of cognition/memory tasks, sleep, and neurological disorders, such as Alzheimer's disease, epilepsy, and Parkinson's disease. Furthermore, we highlight the implication of CFC in the context and for the optimization of invasive and noninvasive neuromodulation and rehabilitation. Mainly, CFC could support advancing the understanding of the neurophysiology of cognition and motor control, serve as a biomarker for disease symptoms, and leverage the optimization of therapeutic interventions, e.g., closed-loop brain stimulation. Despite the evident advantages of CFC as an investigative and translational tool in neuroscience, further methodological improvements are required to facilitate practical and correct use in cyborg and bionic systems in the field.

## Introduction

Cyborg and bionic systems (CBS) focus on the integration of organic and biomechatronic components, with the aim of either restoring lost function or normalizing disease symptoms. Examples of such techniques may include brain–computer interfaces or neuromodulation technologies [e.g., deep brain stimulation (DBS)] [1,2].

Targeting a reliable set of biomarkers is crucial for the development of a useful CBS [3]. Electrophysiological systems such as the brain or heart generate oscillatory activity over a spectrum of frequencies. System outputs such as movement or cognitive process reflect a complex and nonlinear integration of oscillatory neural population activity [4]. This can be accessed using a range of approaches including invasive local field potential or electrocorticogram recordings, or non-invasive measures with either electroencephalography (EEG) or magnetoencephalography.

Multiple neural oscillations across temporal and spatial scales participate in neural information processing [5,6]. In general, low-frequency oscillations are thought to control long-range synchronization, while high-frequency oscillations (HFOs) are believed to be linked to local computation [7]. The question of how these neural oscillations contribute to top-down neural transmission has raised great interest [8,9]. Oscillatory neural activities in multiple frequencies are modulated during a range of tasks (e.g., cognitive tasks) [10–12]. Furthermore, brain stimulation techniques that entrain (or alter) oscillatory activity are in turn known to impact task performance [13,14]. This has led to the belief that oscillatory neural population activity has a causal impact on behavior [15]. In keeping with this, it is also becoming increasingly apparent that neurophysiological oscillations may serve as a biomarker for pathophysiological states such as Parkinson's disease [16].

One particular type of oscillatory coupling, known as cross-frequency coupling (CFC), has gained great interest in medicine and neuroscience. CFC characterizes interactions across different frequency rhythms and is modulated during both physiological processing and pathological states, such as spasticity [17–19]. CFC denotes the statistical association between the phase, amplitude, or frequency of 2 rhythms [17]. CFC applied on simultaneous recordings from different cortical areas reveals a coordinated information exchange in cognitive, sensory, and motor events from long distance to local computation [17]. There are 4 commonly studied types of CFC: phase–amplitude coupling (PAC), amplitude–amplitude coupling (AAC), phase–frequency coupling, and phase–phase coupling [7].

**Fig. 1.**
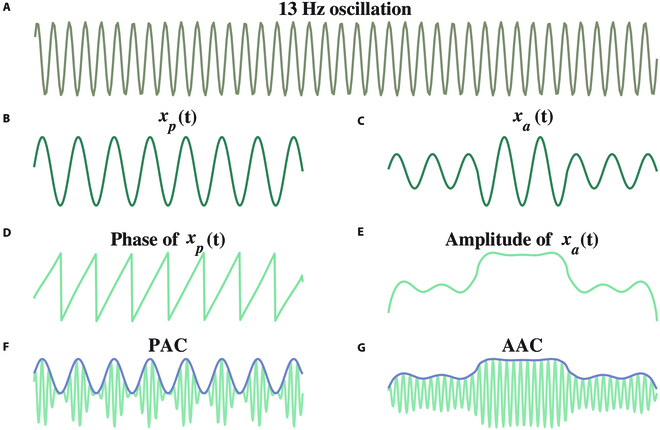
Concepts of phase–amplitude coupling (PAC) and amplitude–amplitude coupling (AAC). (A) High-frequency oscillation (13 Hz). (B) Low-frequency oscillation (2.5 Hz). (C) Low-frequency oscillation with varying amplitude modulations. (D) Phase of a 2.5-Hz oscillation. (E) Envelope of *x_a_*(*t*) oscillation. (F) Oscillatory coupling formations of PAC with 2.5-Hz phase shown in (D) modulating 13-Hz amplitude shown in (A). (G) Oscillatory coupling formations of AAC with 2.5-Hz amplitude shown in (C) modulating 13-Hz amplitude shown in (A).

PAC [20–22] and AAC [23,24] attracted much attention for their association with physiological processing and pathological states. These 2 types of CFCs are depicted in Fig. 1. Figure 1F shows a simulation of PAC, where the activity at 13 Hz is modulated by the phase of a 2.5-Hz wave, forming a nested structure, i.e., *X_PAC_*(*t*) = (*x_p_*(*t*)+1)·sin(2*π*×13*t*), where *x_p_*(*t*) = sin(2*π*×2.5*t*). On the other hand, AAC refers to when the amplitude of the 13-Hz activity is modulated by the envelope of a 2.5-Hz wave (Fig. 1G). In this case, the form of the resulting signal is *X_AAC_*(*t*) = |*H*[*x_a_*(*t*)]|·sin(2*π*×13*t*), where *H*[·] represents the Hilbert transform (HT) of a signal *x_a_*(*t*) = *V*·sin(2*π*×2.5*t*), and *V* is a time-varying variable (Fig. 1B). Both *X_PAC_*(*t*) and *X_AAC_*(*t*) formulated here refer to 13-Hz activities, and both the instantaneous phase (Fig. 1D) and amplitude (Fig. 1E) are derived from the HT of the inputs.

This review seeks to offer a comprehensive overview of the latest developments in CFC research, with a special focus on methodologies, neural mechanisms, and potential applications in CBS, clinical interventions in particular. Firstly, the review will commence by defining CFC and summarizing the current state of knowledge regarding its methodological advances. Next, we will summarize the latest studies on CFC in cognitive processes, and various neurological disorders, including but not limited to Alzheimer's disease, epilepsy, and Parkinson's disease, plus discussions over the potential neuromodulation techniques for clinical interventions. Lastly, the review will consider the challenges and opportunities for the integration of CFC technology into CBS, with future trends in this field being highlighted.

## Methodological Considerations in CFC

CFC provides an approach to encode multiple bodies of CBS. Specifically, the slow wave encodes temporal information via phase coding, while the fast oscillation reflects rhythmic spiking activity [7]. Many methods had been introduced for the computation of CFC. Traditionally, linear approaches were applied to quantify CFC [25–27]. Considering findings suggesting that CFC is prone to dynamic fluctuations [20,28,29], methods for computing time-varying CFC are required.

Aru et al. [30] reported risks of bias in measuring CFC along with several recommendations to evaluate the reliability of different CFC methods. Indicators to evaluate CFC methodologies are summarized as follows: (a) The bandwidth of the extracted decomposition should adequately cover its riding wave (i.e., amplitude modulation). (b) The effect of oscillatory nonlinearity on coupling strength, authentic waveform characteristics, and possible harmonics needs to be carefully validated. (c) The accuracy of the quantitative approach in calculating the instantaneous phase/amplitude modulations also matters. (d) Preserving input-related nonstationarity is important. (e) Either healthy control or surrogate data are needed. (f) Sustaining temporal structure and transient coupling is a necessity.

Extracting a broad range of phase and amplitude modulations from electrophysiological oscillations is crucial for assessing CFC [30,31]. The traditional Fourier transform may result in harmonic artifacts with a loss of information [23,32–34]. Empirical mode decomposition (EMD)—a method of adaptive decomposition [35]—has been proven to capture nonlinear/nonstationary features of irregular patterns more effectively than the Fourier transform. EMD decomposes data into intrinsic mode functions (IMFs) at different frequencies. IMFs are considered promising for calculating CFC [23,36]. However, the sifting process of EMD may result in intermittent patterns at different frequency ranges being mixed within the same IMF (i.e., mode mixing). Therefore, several advanced methods have been proposed for the calculation of CFC. Ensemble EMD-based PAC eliminates the mode mixing phenomenon by iteratively adding Gaussian white noise to ensure refined scale in phase/amplitude-given components [37]. This leads to increased computational complexity. The proposal of masking PAC is computationally efficient and resolves the trade-off between nonlinearity and frequency specificity [38,39]. Recently, variational mode decomposition-derived PAC estimation techniques have been proposed. To avoid spurious couplings caused by dyadic filter banks or harmonics, PACs between irregular oscillators around preferred center frequencies are measured [40].

Traditionally, the HT has been used to calculate phase and amplitude modulations. PAC can subsequently be computed by using metrics such as the modulation index (MI). One well-known method to quantify MI is to measure the nonuniformity of the distribution of the averaged high-frequency amplitude over the low-frequency phase bin. Precisely, a probability distribution, *P* of the high-frequency amplitudes at each low-frequency phase can be constructed. This observed distribution can be compared to a uniform distribution (which would imply no relationship between phase and amplitude) using information theoretic measures such as the Kullback–Leibler (KL) distance. The KL distance can then be normalized by considering the maximum possible entropy, resulting in MI values ranging from 0 to 1.

Tort et al. [41] compared 8 different PAC indicators and concluded that MI had the best performance. PAC can also be estimated through the phase-locking value (PLV) [42] or synchronization index (SI) [43]. Some studies used mean vector length (MVL) as a measure to assess the dependence between phase and amplitude time series by clustering complex vectors [44,45]. Penny et al. compared PLV [46], MVL [44], and envelope-to-signal correlation (ESC) [47] with the general linear model and concluded that all methods comparably performed with suitable conditions (e.g., long epoch with less noise contamination). Moreover, a growing number of toolboxes are devised to calculate CFC, wherein some have relied on Matlab such as Fieldtrip [48] and Brainstorm [49], and others are Python-based toolboxes such as pactools and Tensorpac [50]. These tools support multiple CFC measures and statistical analyses to obtain a corrected CFC. However, the common use of linear analyses could more or less result in spurious couplings.

**Fig. 2.**
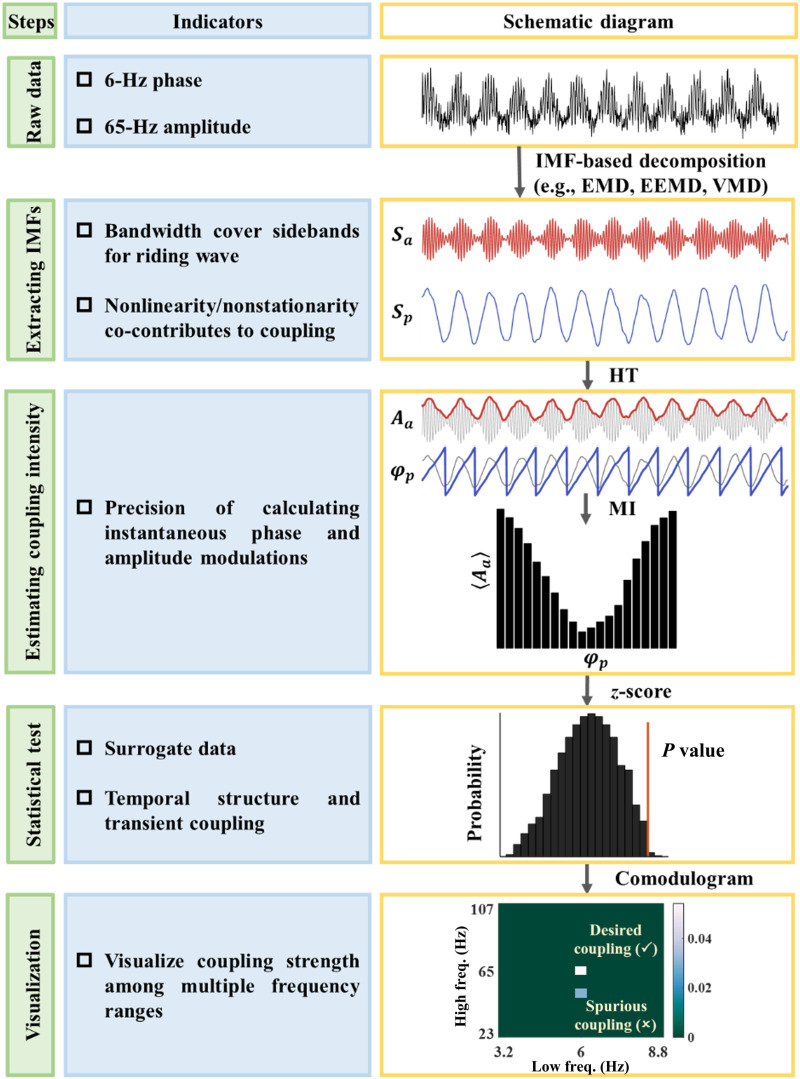
Demonstrating methodology of CFC between 6-Hz phase and 65-Hz amplitude modulations. The left panels show the 5 steps to calculate CFC. The middle panels summarize the indicators of each step to guarantee a reliable CFC. The right panels present schematic diagrams of each step. Firstly, a raw signal with a 65-Hz amplitude modulated by a 6-Hz phase is illustrated. Secondly, all phase-given *S_p_* and amplitude-given *S_A_* IMFs are calculated, wherein all IMFs are extracted by IMF-based decompositions. Next, the instantaneous phases *φ_p_* and envelopes *A_a_* of the corresponding IMF are obtained by HT with MI serving as a measure of coupling strength. After that, surrogate data are created to access the significance of MI. Lastly, a cross-frequency comodulogram is adopted to display coupling strength across multiple frequencies. The white block denotes the desired coupling between the 6-Hz phase and 65-Hz amplitude, while the blue block represents a spurious coupling.

After the decomposition procedure, the standard process to calculate coupling strength is as follows. Illustrated by the case of PAC, either cycle-by-cycle frequencies or instantaneous ones are applied to eliminate the effects of intra-wave variation per decomposition. To obtain the cycle-based frequencies of the *i*th decomposition of the *j*th channel recording, the phase series is unwound, then the expansion phase series at points in time spanning 2*π* integer increments is identified to generate a cycle-by-cycle conversion. The cycle-based frequency can be approximated by a secant to the instantaneous frequency [36,37]. Here, *i* = 1, 2, …, *N*, where the value of *N* represents the total number of IMFs. Meanwhile, the phase/amplitude modulations derived from the HT of all selected decompositions across montages of interest are used to assess the cross-channel and/or cross-decomposition coupling intensity [11,41]. The statistical significance of the MI can be tested by generating surrogate data from individual MI-contributed decompositions using a bootstrap strategy in a cycle base [51,52]. The average and standard deviation of the permuted MIs are used to determine the *z*-score of the original MI, and a significance threshold (*α* = 0.05) can then be applied. A cross-frequency comodulogram can then be used to visualize coupling strengths. Figure 2 shows the methodology of CFC exemplified by a signal with a 6-Hz phase modulating a 65-Hz amplitude. Table 1 lists the bibliography of “methodological advances of CFC”.

**Table 1.** Bibliography of methodological advances of CFC.

Steps	Ref.	Key points
Accessing phase and amplitude modulations	Aru et al. [30]	Some potential risks of bias in measuring CFC are summarized.
	Kramer et al. [32]	Fourier analysis may result in harmonic artifacts.
	Colgin et al. [26]	The wavelet technique was applied to quantify CFC.
	Pittman-Polletta et al. [36]	The integration of EMD enables extracting nonstationary and nonlinear broadband rhythms in calculating PAC.
	Shi et al. [37]	Ensemble EMD-based PAC eliminates the mode mixing phenomenon.
	Yeh and Shi [38]	Masking PAC is computationally efficient and resolves the trade-off between nonlinearity and frequency specificity.
	Zhang et al. [40]	Variational PAC avoids occurrence of spurious couplings due to dyadic filter banks or harmonics.
Estimating coupling intensity	Tort et al. [41]	MI exhibited superior performance among 8 different PAC indicators.
	Penny et al. [42]	PLV was originally developed to quantify phase synchronization between trials.
	Cohen [43]	SI was presented to test CFC between upper *θ* and *γ*.
	Canolty et al. [44]	Coupling strengths during cognitive processes were assessed by MVL.
	Bruns and Eckhorn [47]	ESC was proposed to measure correlations between different bands.
Statistical analysis	Pittman-Polletta et al. [36]	To sustain the temporal structures, surrogate data with cycle-shuffled amplitude and phase were generated.
Visualization	Yeh et al. [23]	Redistributing CFC across a frequency scatter plot based on the cycle-by-cycle frequencies corresponding to each IMF pair.

## Progress of Electrophysiological Couplings in Physiology and Neuroscience

**Fig. 3.**
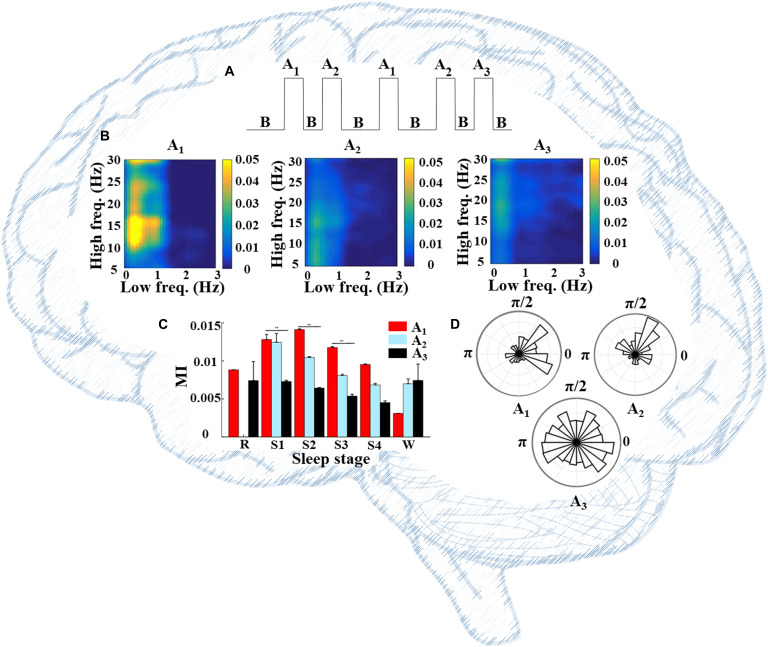
PAC associating various brain rhythms and varying by physiological states. (A) CAP consists of A and B phases, in which A contains 3 subtypes including A_1_, A_2_, and A_3_. (B) PAC comodulograms differ by phase-A subtypes, of which A_1_ shows stronger *α*/low *β*-amplitude-related PACs. (C) Significant differences (*P* < 0.0001) of *δ*-*α*/low *β* PACs among phase-A subtypes in all sleep stages except S4 were shown. (D) The distribution of phase difference between *δ* phase and *α*/low *β* amplitude is displayed in the polar histogram chart. Subtype A_3_ showed a relatively disperse distribution compared to the 2 other subtypes.

Information flow typically involves multiple sites of specialized processing [53]. CFC can provide a framework for both local and distributed information processing within neural networks, thereby serving the coordination of neural oscillations over multiple spatial scales [17,54–61]. Owing to this, disturbances of information processing in certain neurological disease states may be inferred through the observation of changes in CFC relative to healthy control populations [62,63]. In general, past findings have shown that stronger coupling tends to occur with higher neural computational needs. For example, during sleep, PAC coordinates various brain rhythms and varies across cyclic alternating patterns (CAPs) (Fig. 3). CAPs are the periodic pattern of sleep comprising A and B phases, in which A contains 3 subtypes including A_1_, A_2_, and A_3_ (Fig. 3A). Past findings suggest that *δ*-*α*/low *β* PAC is stronger in subtype A_1_ (Fig. 3B) than in the 2 other subtypes. This strong coupling may regulate sleep structure and preserve working memory [38]. The relatively disperse distribution of phase differences between *δ* phase and *α*/low *β* amplitude in A_3_ supports a weaker *δ*-*α*/low *β* PAC (Fig. 3D). Table 2 shows the bibliography of the “progress of CFC in neuroscience and physiology”.

**Table 2.** Bibliography of the progress of CFC in neuroscience and physiology.

Ref.	Disease	Subtype of CFC	Key points
Axmacher et al. [10]	Pharmacoresistant temporal lobe epilepsy	*θ*–*γ*	*θ*–*γ* PAC exhibited greater prominence during cognitive tasks.
Rajji et al. [71]	Healthy	*θ*–*γ*	PAS increases *θ*–*γ* PAC in the dorsolateral prefrontal cortex.
Mondragón-Rodríguez et al. [75]	Alzheimer's disease	*θ*–*γ*	A reduction in *θ*–*γ* PAC between the hippocampus and prefrontal cortex indicates early cognitive impairments.
Etter et al. [81]	Alzheimer's disease	*θ*–*γ*	Optogenetic stimulation can restore memory performance and hippocampal *θ*–*γ* PAC.
Turi et al. [83]	Healthy	*θ*–*γ*	Stimulations at the *θ*–*γ* frequency over the trough impaired cognitive control.
Amiri et al. [97]	Mesiotemporal lobe epilepsy	*δ/θ*-HFO	Interictal HFOs in the SOZ were modulated by *δ/θ* phase.
Ma et al. [102]	Frontal lobe epilepsy	*δ*–*β*/*γ*	Strong *δ*–*β*/*γ* PAC emerges around the SOZ during pre-seizure periods.
de Hemptinne et al. [128]	Parkinson's disease	*β–γ*	*β* phase waveform in both the primary M1 and STN modulate broadband-*γ* amplitude in M1.
de Hemptinne et al. [129]	Parkinson's disease	*β–γ*	STN DBS can reduce *β–γ* coupling.
He et al. [136]	Parkinson's disease	Gait phase-*α*/*β*	Gait phases associated modulations of *α*/*β* band activity in PPN.
Jin et al. [122]	Parkinson's disease	*δ/θ*-gait-related *β*	Gait-related *β* amplitude is driven by lower-frequency components modulated by auditory stimuli.
Yin et al. [137]	Parkinson's disease	*β–γ*	Increased PAC in MI indicates higher probabilities of gait problems.
Muthuraman et al. [143]	Parkinson's disease	*γ-*stimulation frequency	The presence of CFC suggests that DBS utilizes clinically effective frequencies to induce intrinsic FTG oscillations through a mechanism of entrainment.

### The role and potential of CFC as a biomarker for cognitive and memory tasks

CFC is believed to play a special role in regulating performance in cognitive and memory tasks. Oscillations in both the *θ* (5 to 8 Hz) and *γ* (30 to 150 Hz) bands display modulations in such tasks. Studies in rodents have reported that a strong coupling between *θ* and *γ* activities emerges when decision-making or learning tasks are performed [64,65]. Strong *θ*–*γ* PAC also emerges within the hippocampus during the performance of context-learning tasks [51]. Similarly, *θ*–*γ* CFC is considered to play a crucial role in cognition and memory in humans [10,46,55,66,67]. A recent study revealed that the extent of *θ*–*γ* CFC negatively correlated with the development of mild cognitive impairment [68], hence suggesting that reductions in *θ*–*γ* coupling may relate to degenerative pathologies.

One reported transcranial magnetic stimulation (TMS) paradigm for inducing neuroplasticity is paired associative stimulation (PAS). One recent study showed that PAS can increase *θ*–*γ* PAC within the dorsolateral prefrontal cortex (DLPFC; an area of the frontal lobe that is crucially involved in executive functions such as working memory [69]), suggesting that PAC could be a potential indicator of neuroplasticity [70]. *θ*–*γ* PAC was reported by Rajji et al. [71] to support the organization of information in N-back working memory tasks. Soto and Jerbi [72] observed a clear decrease in *θ*–*γ* PAC during N-back trials that did not involve information ordering. Closed-loop auditory stimulation was reported to locally modulate *δ-α/*low *β* coupling in the frontal area. This modulation could potentially be utilized to influence neuroplasticity that occurs during sleep in the targeted brain network [73].

The diagnosis of common neurodegenerative disorders such as Parkinson's disease (PD) or Alzheimer's disease (AD) can only be confidently made once suggestive clinical signs and symptoms are present. It is well recognized, however, that these conditions often have a prodrome [74], which can be many years long. PAC may serve as a biomarker of prodromal states of neurodegeneration. For instance, in AD, an early sign of neuronal dysfunction leading to cognitive impairments could be a reduction in *θ*–*γ* coupling between the hippocampus and prefrontal cortex [75,76]. This could be relevant in terms of identifying at-risk populations and trialing both pharmacological and non-pharmacological neuroprotective therapies [77,78]. Interestingly, *θ*–*γ* coupling has been reported to be enhanced by the use of CBS [79,80]. For example, Etter et al. [81] used optogenetic stimulation to restore memory performance and hippocampal *θ*–*γ* PAC in a mouse model of AD. Transcranial alternating current stimulation (tACS) has been recently shown to modulate top-down control and functional connectivity (*θ*–*γ* coupling) across the frontal-occipital regions, leading to enhanced performance in working memory tasks [82]. Of note, stimulations at the *θ*–*γ* frequency over the trough have been found to impair cognitive control [83]. Hence, it could theoretically be possible to develop a closed-loop cognitive rehabilitation training set, integrated with techniques/paradigms such as PAS and cognitive exercise. Such a system is designed to increase *θ*–*γ* PAC feedback in the hippocampal/cortical regions to favor memory consolidation.

### Coupling to facilitate the diagnosis and treatment of epilepsy: Biomarkers for seizure onset and non-pharmacological treatment options

HFOs may serve as a biomarker of the epileptogenic zone (EZ) or seizure onset zones (SOZs) [84,85]. Although HFOs occur more commonly within the SOZ/EZ than in other brain areas [86–89], they can also be generated by the nonepileptic somatosensory or motor cortices at rest or during movement [90–93]. Therefore, the application of HFO recordings for guiding epilepsy surgery resection margins has been limited [94–96].

Many studies have reported that interictal HFOs in the SOZ are modulated by the slow-wave phase [97–100]. Ibrahim et al. [101] showed that PAC between HFO amplitude and *θ/α* phase was significantly higher in the SOZ than in other cortical regions. Also, several studies exploring CFC in epilepsy found that *δ*–*γ* PAC could be a promising biomarker for locating the SOZ/EZ [98,102]. For frontal lobe epilepsy, it has been observed that a prominent *δ*–*β*/*γ* PAC occurs around the SOZ during pre-seizure periods [102]. This would support a role for PAC in regulating seizure onset [103]. Interestingly, Guirgis et al. [104] observed that the presence of *δ*-modulated HFOs provided a satisfactory indicator of the resection margin of an EZ.

For some patients with drug-resistant epilepsy, the removal of brain tissue is not advisable due to the presence of multiple seizure foci or fears of postoperative functional deficits [105]. Developing sophisticated neural regulatory techniques, for instance, vagus nerve stimulation (VNS ®, LivaNova, Inc.) or responsive neurostimulation (RNS ®, NeuroPace, Inc.), has been a recent focus [106,107]. VNS and RNS were approved as non-pharmacological treatments for focal epilepsy by the U.S. Food and Drug Administration in 1997 and 2014, respectively. These neurostimulation devices deliver electrical stimulation with adjustable parameters to reduce the frequency of seizures [108]. The RNS system is designed as a closed-loop device that delivers electrical stimulation immediately upon recognizing possible electrocorticogram seizure activity [109,110]. Meanwhile, the VNS with the recently released generator model 106 AspireSR® can trigger simulation automatically based on the increased heart rate, which may indicate seizures [111]. Randomized trials using VNS and RNS have demonstrated the effectiveness of neuromodulation—with a responder rate of up to 50% [112–114]. However, the mechanisms of neurostimulation remain unclear, and this hinders the development of further improvements in this technology.

### Movement-related neurophysiological basis and neuromodulation techniques for Parkinson's disease rehabilitation

**Fig. 4.**
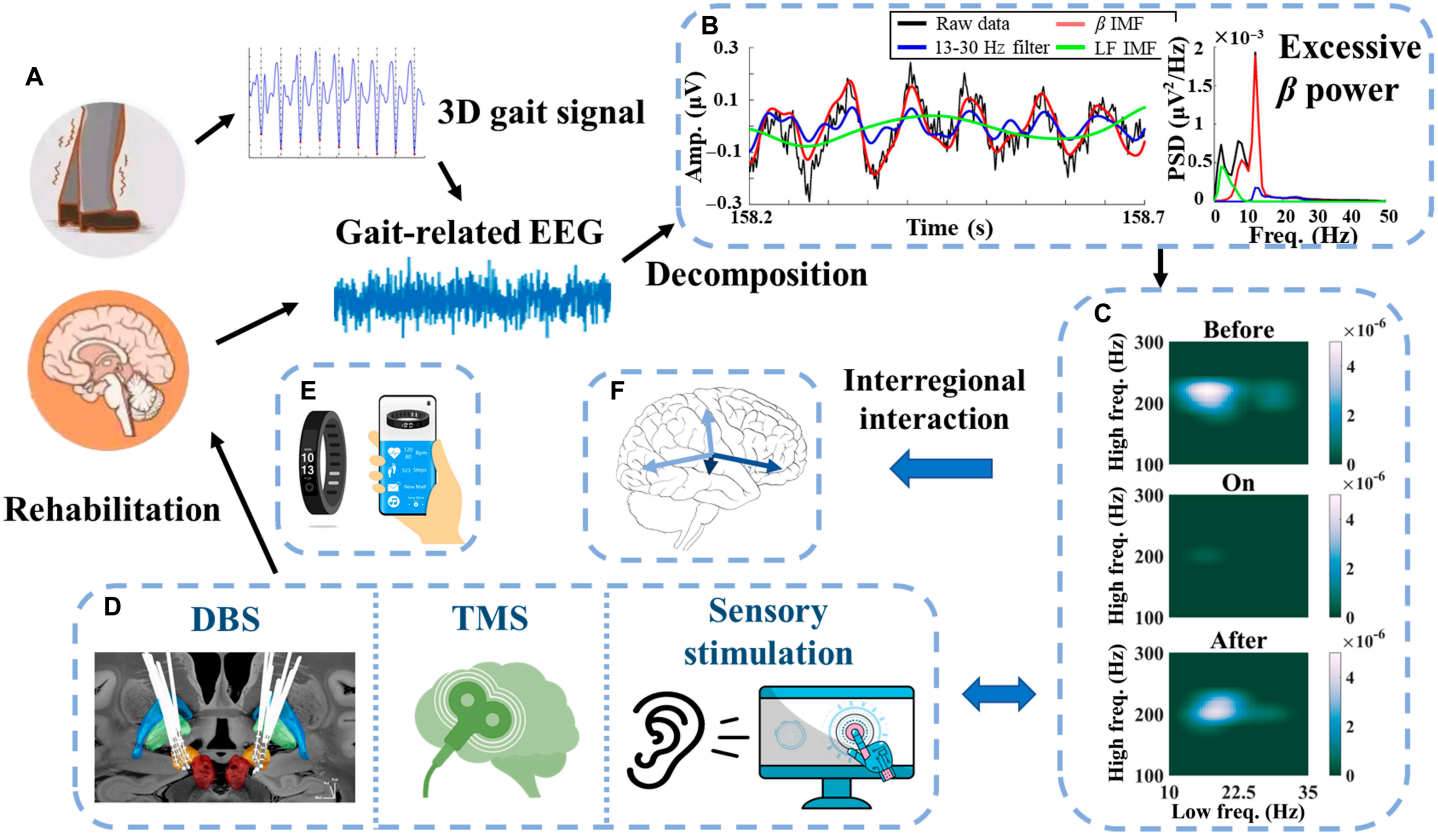
An example of applying CFC with multiple neuromodulations to rehabilitation interventions for individuals with Parkinson's disease. (A) A 3-dimensional gait signal and gait-related EEG of a PD patient with motor impairment. (B) After signal decomposition, excessive *β* power emerged in basal ganglia. (C) Excessive PAC between *Lβ*/*Hβ* and HFO in the STN was observed in a patient with PD, as shown in the left panel, which was suppressed by stimulation (middle panel), with a subsequent rebound after the stimulation was removed (right panel). (D) Neuromodulations, such as DBS, TMS, and sensory stimulation. (E) The varying PAC with stimulation provides feedback to the neuromodulation paradigm. (F) PAC results reflect the interregional interaction/communication in the brain.

Motor impairment in PD is characterized by excessive synchronization in the *β* band within the basal ganglia (BG) [115,116] (Fig. 4B). Additionally, *β* power can be suppressed with both dopaminergic medication and stimulation and the extent of treatment-related suppression correlates with treatment-related clinical improvements in bradykinesia and rigidity [117–122], hence suggesting an important pathophysiological role of *β* oscillations. *γ* oscillation, in contrast, synchronizes strongly in the BG and thalamus at the initiation of contralateral movements [123,124]. Movement-related broadband *γ* synchrony (30 to 100 Hz) increases excitability between periods of inhibition, supporting interregional interaction/communication [125,126]. Note that the occurrence of narrowband *γ* activity (60 to 90 Hz) induced either by levodopa or DBS is linked to dyskinesia [127]. Studies have suggested that cross-frequency interactions may have a marked impact on BG information processing in PD [119,120]. The phase of the *β* waveform within both the primary motor cortex (M1) and the subthalamic nucleus (STN) has been shown to modulate broadband-*γ* amplitude in M1 in PD [128–133]. This cortical *β*–*γ* coupling may also be suppressed by STN DBS.

Studies exploring the relationship between oscillatory activity and gait have revealed that low-*γ* frequency oscillations in the motor cortex are modulated by the gait phase [134] and central midline sites [135]. In PD, gait phases are known to associate with modulations of *α*/*β* band activity within the pedunculopontine nucleus (PPN) [136]. Our past work has also shown that *β* band modulation within the STN is time-locked to contralateral steps in PD [121]. High-*β* synchronization is suppressed when the contralateral foot is raised and displays a rebound following a heel strike. Recently, we observed that these gait-related *β* frequency amplitude modulations could themselves be driven by lower-frequency components that can be modulated by auditory stimuli [122], hence providing a neurophysiological substrate for a link between auditory and motor processing. This can be partially explained by the fact that elevated PAC in PD may result in greater (cortical) processing demands, which, in turn, could contribute to gait problems like freezing episodes [137]. Studies such as this provide insights into the cortical circuits that need to be modulated to target specific disease symptoms. The inhibitory circuit formation in the cortex of PD patients is more strongly inhibited in response to stimulation than normal individuals, indicating that the occurrence of cortical disinhibition could be an early, and possibly prodromal, characteristic of PD [138]. Pezzopane et al. [139] reported targeting *θ*–*γ* tACS to short intracortical inhibition through stimulation resulted in a decrease in inhibition following the stimulation.

Neuromodulation techniques for PD can either be invasive or non-invasive. Noninvasive tools include TMS, transcranial direct current stimulation, and tACS, while the most frequently employed invasive technique is DBS [140]. High-frequency DBS (at frequencies of around 130 Hz) can suppress increased BG *β* activity and ameliorate symptoms such as bradykinesia or rigidity [141]. While conventional continuous DBS is already established, a promising next-generation DBS technique is closed-loop DBS, in which the delivery of stimulation is titrated based on a neurophysiological biomarker [142]. For example, the CFC observed between *γ* oscillations and the volume of tissue-activated power at stimulation frequency in Muthuraman et al.'s report [143] indicates that DBS as per clinically effective frequencies may induce intrinsic FTG oscillations through an entrainment mechanism. Closed-loop DBS has attracted a growing amount of attention for the treatment of PD [144]. Gilron et al. reported the effectiveness of adaptive DBS with the Summit RC+S (Medtronic) device. This adjusts stimulation parameters based on the detection of neurophysiological activity that may relate to particular symptoms such as dyskinesia [142]. A recent study showed that within a gait cycle, significant positive correlations were observed between low *β* power and gait muscle activities that can be used to forecast the gait events and freezing episodes [145]. This also supports the use of closed-loop neuromodulation therapies that can be controlled through specific commands.

Thinking about rehabilitation approaches, robot-assisted devices such as the Tymo system (Tyromotion, Austria) have advantages over traditional physiotherapy (e.g., stretching and muscle strengthening) for impacting gait. These devices enable the task-oriented design of exercises and adjustment of the intensity of exercises [49,146–148].

Figure 4 illustrates how CFC biomarkers can be applied to several neuromodulation or rehabilitation techniques. As per the 3-dimensional gait signal, the gait-related EEGs from a PD patient with motor impairment are extracted (Fig. 4A). Excessive *β* power is observed in BG after signal decomposition (Fig. 4B). Obvious *Lβ*/*Hβ-*HFO PAC emerges in the STN (left panel in Fig. 4C), while stimulation techniques, e.g., DBS, TMS, and sensory stimulation (Fig. 4D), can suppress the abnormal PAC (middle panel in Fig. 4C), with a subsequent rebound after the stimulation is removed (right panel in Fig. 4C). The varying PAC under stimulation provides feedback to the neuromodulation paradigm (Fig. 4E). The PAC dynamics support the interregional interaction/communication in the brain (Fig. 4F), which facilitates the understanding of the pathological neural network.

## Conclusion and Future Outlook

This review focuses on the methodologies, mechanisms, and applications (neuro-control and rehabilitation treatment) of CFC in neuroscience and medicine. Reliable CFC enables characterizing multi-frequency interactions and reflects how these coupled oscillations contribute to top-down neural transmission. CFC, PAC in particular, provides relatively precise metrics of entangling temporal structure in neural circuits and is, hence, linked to both motor and cognitive function in healthy and diseased states, which manifests that it holds great promise in serving as an electrophysiological feature to inform real-time neuromodulation. In addition to their use in measuring cognitive and motor states, CFC metrics may also allow for the monitoring of disease progression and therapeutic responses, supporting clinicians and scientists to allocate brain regions and temporal periods with deteriorated neural functions.

Referenced to other biomarkers, such as band power [149], evoked compound action potential [150], and abnormal synchrony [151], CFC may offer a deeper understanding of the underlying entangling oscillatory mechanisms of neurological disorders. By identifying the interested CFC patterns that are disrupted by changing physiological or pathophysiological status, researchers can gain insight into the affected neural circuits, which could facilitate the development of more targeted and effective interventions.

We have discussed some common pitfalls to consider when computing CFC metrics, including techniques to avoid the detection of spurious CFC. The criteria proposed by Aru et al. can serve as quality control to avoid methodological confounds. The cycle-based permuted nonlinear approaches introduced in this review suggest a feasible path toward a more reliable CFC estimation.

Although CFC has been greatly developed, several challenges to implementing CFC in cyborg and bionic systems need to be addressed to facilitate translating CFC findings into these systems. One crucial challenge is the development of reliable and effective CFC measurement techniques to integrate into a CBS, relying on sensors and devices to real-time access brain activities, followed by translating them into control signals for prosthetic limbs, etc. Thus, developing promising algorithms to evaluate CFC intensities without introducing clear delays or bias is highlighted next. Successful translation of CFC findings into a CBS requires precise decoding of electrophysiological recordings and then translating CFC biomarkers into controls of CBS devices. Such algorithms and controls require minimizing time lags and false alarms in real use. Lastly, these translation uses require rigorous testing and validation to ensure their safety, effectiveness, and reliability in real-world applications. These involve extensive testing and validation in preclinical and clinical settings, along with continued monitoring and optimization of CFC-based CBS over time.

Although many challenges remain, including the handling of brain stimulation artifacts and real-time deployment, robust algorithms and controls, and rigorous testing and validation of these systems in real uses, there is much to be optimistic about regarding the therapeutic deployment of CFC-based CBS.

## Data Availability

Data of this paper are available by emailing chien-hung.yeh@bit.edu.cn.
